# In Vitro Evaluation of Tooth Enamel Abrasion and Roughness Using Toothpaste with and Without Activated Charcoal: An SEM Analysis

**DOI:** 10.3390/dj13100482

**Published:** 2025-10-21

**Authors:** Fiorella Thais Aquino Carmen, Renzo Jesús Pro Romero, Alexander Roger Espinoza Salcedo, Paul Martín Herrera-Plasencia

**Affiliations:** Facultad de Ciencias de la Salud, Escuela Profesional de Estomatología, Universidad César Vallejo, Piura 20009, Peru; ftaquino@ucvvirtual.edu.pe (F.T.A.C.); rjproro@ucvvirtual.edu.pe (R.J.P.R.); aespinozasal@ucvvirtual.edu.pe (A.R.E.S.)

**Keywords:** activated charcoal, dental abrasion, dental enamel, toothpastes

## Abstract

**Background/Objectives:** Dental enamel constitutes the first barrier of defense against external factors that constantly generate wear and damage. This study aimed to evaluate in vitro the abrasion and roughness of dental enamel using toothpaste with and without activated charcoal and to analyze this under scanning electron microscopy (SEM). **Materials and methods**: The research design was experimental; 10 enamel blocks were randomly assigned to each group to perform brushing cycles with soft- and medium-filament brushes with two types of toothpaste, one with activated charcoal and one without activated charcoal. A pumice stone with etching acid was used as the positive control and artificial saliva served as the negative control; both were analyzed separately. Roughness was evaluated using a roughness meter and abrasion with an analytical balance. The surface of the enamel blocks of each group was randomly analyzed under an SEM. Statistical analysis was performed using the Shapiro–Wilk test and the homogeneity of variances with Bartlett’s test. Student’s *t*-test (two-tailed) was applied to compare tooth enamel roughness and abrasion. **Results:** Both enamel roughness (*p* = 0.0016) and abrasion (*p* = 0.0001) were significantly higher in the groups using activated charcoal paste and medium-filament brushes. SEM observation revealed greater alteration on the surface of the enamel subjected to brushing cycles with activated charcoal paste and a medium-filament brush. **Conclusions:** The in vitro study showed that the use of toothpaste with activated charcoal increases the roughness and abrasion of tooth enamel, especially when the medium-filament brush is used.

## 1. Introduction

Tooth enamel, the hardest and outermost tissue of the tooth, is constantly exposed to mechanical, chemical, and biological factors that can progressively compromise its surface integrity. These factors include biofilm and acids produced by bacteria, food, and pigmenting substances, as well as physiological factors such as chewing or pathological mechanisms such as abrasion, erosion, or attrition, which cause the accelerated loss of its structure [1,2]. Effective plaque removal is essential for maintaining optimal oral health and, for this purpose, toothbrushes and toothpastes are used. However, inappropriate use or overuse of these products can lead to alterations in the enamel surface, such as roughness and abrasion [3,4].

This wear can be exacerbated by improper brushing techniques, excessive force, or the use of abrasive toothpastes [5]. Several studies have shown that aggressive brushing and the use of medium-bristle toothbrushes can increase enamel wear, especially when combined with abrasive agents such as activated charcoal. In addition, many of these formulations lack fluoride, which reduces their anti-caries and remineralizing capacity [6]. In contrast, conventional toothpastes with calibrated abrasives such as silica and fluoride offer a better balance between efficacy and safety.

Dental abrasion is a multifactorial phenomenon caused by friction on tooth surfaces and contact with abrasive substances, resulting in the loss of mineral material. This wear can lead to the exposure of the dentinal tubules, increasing tooth sensitivity and even causing tooth loss [7,8,9,10]. On the other hand, surface roughness also plays an important role in the health of dental enamel, favoring the adhesion of microorganisms and promoting the accumulation of plaque, which can lead to problems such as gingival inflammation and the formation of caries [11,12]. Factors such as poor brushing technique, the amount and type of toothpaste, the frequency of brushing, the hardness of the toothbrush filaments, and the pressure exerted during tooth cleaning generate abrasion and roughness of the enamel, causing significant changes in its surface [13].

In recent years, the use of activated charcoal in personal care products, especially in toothpastes, has gained popularity, which has led to a growing interest in its possible effects on tooth enamel abrasion and roughness [6]. Recent studies have shown significant enamel loss in groups using toothpaste with activated charcoal, as well as alterations such as surface roughness [14,15,16]. This trend is partly due to the high percentage of toothpastes containing whitening agents, including activated charcoal. However, despite the widespread promotion of its whitening benefits, the scientific evidence supporting its effectiveness is limited, and many claims about its therapeutic effects are not substantiated [17,18].

In this context, there is a clear need for controlled in vitro studies that objectively evaluate the abrasive effects of activated-charcoal-based toothpastes under standardized conditions. Furthermore, few studies have simultaneously evaluated enamel roughness and abrasion, limiting understanding of their combined impact on tooth surfaces. Given the controlled environment of this in vitro study, the null hypothesis was formulated that there would be no significant differences in enamel abrasion or roughness between toothpastes with and without activated charcoal, nor between the use of soft- or medium-bristle toothbrushes.

The purpose of this research was to evaluate in vitro the abrasion and roughness of dental enamel using toothpaste with and without activated charcoal and to analyze this under scanning electron microscopy (SEM).

## 2. Materials and Methods

### 2.1. Sample Size

The minimum sample size required was calculated using G*Power v.3.1 software. The power obtained, with a sample size of 10 specimens per group, was higher than 99% (Cohen’s between 2.58 and 4.47). This confirms that the sample size used is adequate and sufficient to detect statistically significant differences in abrasion and surface roughness of tooth enamel.

**Figure 1:**
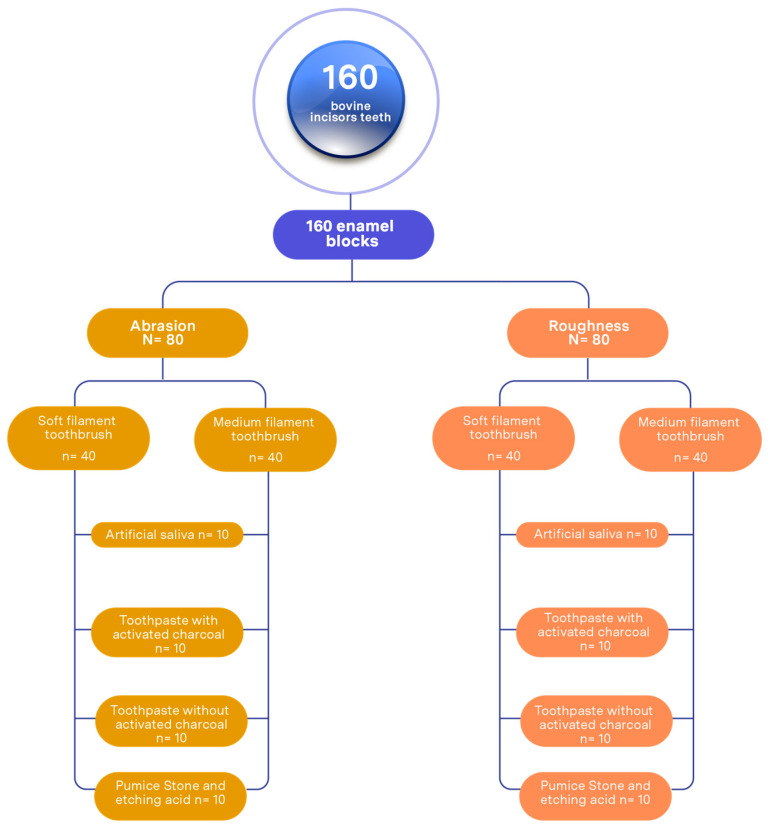
Sample distribution.

A total of 160 enamel blocks were assigned, of which 80 were used for abrasion analysis and 80 for roughness study. Both procedures were performed independently and simultaneously, as detailed below (Figure 1).

Negative control group: 20 enamel blocks immersed in artificial saliva; 10 for the group with soft-filament toothbrushes (SFTs) and 10 with medium-filament brushes (MFTs).

Group brushing with toothpaste with activated charcoal: 20 enamel blocks subjected to brushing cycles, distributed into 10 with soft-filament toothbrushes and 10 with medium-filament brushes.

Group brushing with toothpaste without activated charcoal: 20 enamel blocks subjected to brushing cycles, distributed into 10 with soft-filament toothbrushes and 10 with medium-filament brushes.

Positive control group (pumice stone and etching acid): 20 enamel blocks subjected to brushing cycles, distributed into 10 with soft-filament toothbrushes and 10 with medium-filament brushes.

The selection of toothpastes was based on their commercial availability and popularity in the local market. A toothpaste containing activated charcoal was chosen due to its increasing use in oral hygiene products promoted as whiteners, despite limited scientific evidence regarding its abrasive safety. As a control, a conventional toothpaste without activated charcoal and with standard abrasive characteristics was selected in order to compare the effect of these products on enamel abrasion and roughness.

### 2.2. Data Compilation

The bovine teeth used in this study were obtained after the animals were slaughtered in slaughterhouses authorized for human consumption, without any intervention by the researchers. A total of 160 bovine incisors were collected, donated by the slaughterhouse area of Frigorifico Camal La Colonial, Lima, Peru [19,20]. The teeth were certified by a veterinarian, who determined that they were suitable for the study (Peruvian Veterinary Medical Association: 5121). The present research was of experimental design and approved by the Research Ethics Committee of the Professional School of Stomatology of the Universidad César Vallejo, Piura, Peru (Resolution N° 0089-2023-/UCV/P), dated 16 December 2023. Teeth with intact buccal surfaces were selected, excluding those with crown defects, carious lesions, or fractures. The teeth were extracted using a straight punch and a Rottmann Stainless Steel Milano forceps. Subsequently, they were washed with running water and thoroughly cleaned with a scalpel No. 15 to eliminate organic tissue remains. The blocks were obtained from the vestibular surface of the teeth and cut with a medium-grit diamond disk (NTI Kerr D335-190, Rotary dental Instruments, Kahla, Germany). Each block measured 5 mm × 5 mm × 2 mm, verified using a digital Vernier caliper (Mitutoyo, 200 mm, Kanagawa, Kawasaki, Japan; lot B23082834). The surfaces were polished for 30 s with N° 1200 sandpaper under constant irrigation with water to obtain a uniform surface. A total of 160 enamel blocks were obtained, of which 80 were selected for the roughness study and 80 for the abrasion analysis [16,21,22] (Figure 1).

### 2.3. Materials

The materials used in the study were as follows:

- Toothpaste with activated charcoal: Oral B Natural Essence (Procter & Gamble, Naucapal de Juárez, Mexico, lot 30904354P4). Toothpaste without activated charcoal: Colgate Total 12 Antisarro (Colgate Palmolive, Guanajuato, Mexico, lot 3348MX1131);
- Toothbrushes with soft filaments: Colgate Procuidado (Colgate Palmolive, Binh Duong Province, Vietnam, lot 3333Z). Toothbrushes with medium filaments: Colgate Colors (Colgate Palmolive, Binh Duong Province, Vietnam, lot 0374QZ);
- Artificial saliva (manufactured by LUSA, Laboratorios Unidos S.A. Lima, Peru, lot 2081683);
- Pumice stone: fine grain size (<50 μm). (Manufactured by Vitalloy, Lima, Peru);
- Phosphoric acid 37% (Manufactured by Densell, Buenos Aires, Argentina; lot 2204789).

### 2.4. Procedure

For the abrasion experiment, the enamel blocks were dried for 24 h at 37 °C before and after the brushing cycles using an induction oven equipped with an infrared radiation thermometer (Shenzhen Mestek Electronics Co., Ltd., model IR02B; Shenzhen, China, range −50 °C to 800 °C). The specimens were weighed before (M1) and after (M2) brushing, and the mass loss (Mfinal = M1 − M2) was calculated using a high-precision electronic balance (EHuaZhi PTX-FA210S; HuaZhi Electronic Technology Co., Ltd., Putian, China), accuracy 0.0001 g, serial number 2107214210.

Brushing was performed using automatic brushing equipment (HTL, model YX 3000-280007G; HTL Instruments, Lima, Peru), which simulates standardized linear movements. The protocol consisted of a total of 120 min of brushing, equivalent to 20 days of regular clinical brushing (3 times a day for 2 min per session) [10,23]. The brushes were adapted to the brushing equipment, and each sample underwent six consecutive 15 s sessions, distributed in series that replicated the manual brushing movement.

The brush handle was cut at 5 cm to fit the machine tube and the enamel block was secured on a base that had a bolt applying low pressure. The cycling parameters were 5 Hz frequency and 200 g or 1.9 N load, with rinsing and addition of bean-sized toothpaste to the middle of the brush every 15 min. After the cycling process was completed, it was dried in the induction oven at 37 °C for 24 h and the second weight (M2) was recorded. This procedure was carried out simultaneously, using a new brush for each brushing cycle on the enamel blocks and individually [10,23,24,25].

In the roughness experiment, the enamel blocks were mounted on acrylic discs exposing only the vestibular surface. A roughness meter (Huatec SRT-6200; Huatec Group Corporation, Beijing, China; serial number N921838; resolution 0.001 μm). was used for measurement in microns (μm) before (μ1) and after (μ2) to determine the difference (μFINAL = μ1 − μ2). Simulating brushing with cycling equipment (HTL, model YX 3000-280007G; HTL Instruments, Lima, Peru, approximation of 1 cycle) corresponded to 20 days, with a frequency of 3 times a day, for two minutes (120 min in total) [16,21].

The initial roughness measurement (μ1) of the enamel blocks was recorded, and then the brushing cycle was performed. The cycling parameters were 5 Hz frequency and 200 g or 1.9 N load. A bean-sized amount of toothpaste was added to the middle part of the brush every 15 min and washed with water for 30 s. At the end of the cycling process, the second roughness measurement was recorded [16,21]. The whole process was performed independently and in parallel using a new brush for each brushing cycle on the enamel blocks.

### 2.5. Scanning Electron Microscope (SEM)

**Figure 2:**
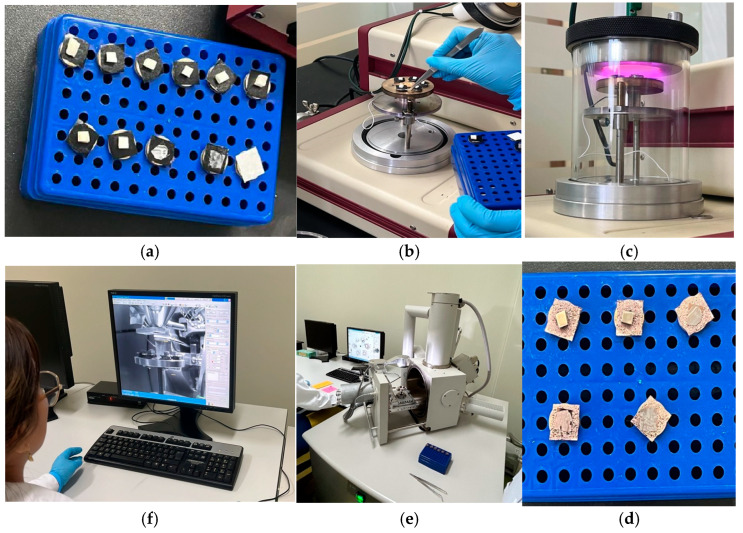
SEM setup. Flow chart for SEM image preparation of bovine dental samples: (**a**) mounting on stub, sample holder with conductive adhesive; (**b**) placement of samples on metallization base; (**c**) gold sputtering, application of metallic coating by sputtering; (**d**) review of correctly metallized samples; (**e**) SEM imaging, insertion into SEM chamber for visualization under vacuum; and (**f**) acquisition and analysis, digital image capture for morphological analysis.

The following samples were randomly selected and analyzed under SEM: one block of untreated enamel, one block of the negative control group, one block of the positive control group, and four blocks subjected to brushing. Of the latter, two were exposed to paste with activated charcoal (one with soft-filament brush and one with medium filaments) and two to pastes without activated charcoal (one with soft filaments and one with medium filaments). The preparation of the blocks was carried out as follows: First, they were dried in the induction oven for 24 h at 37 °C, and then all the enamel blocks were fixed in carbon fiber on metal platforms known as Stubs, metallizing them with gold through a SPI-MODULE brand metallizer (Figure 2). The observation was performed in the scanning electron microscope (FEI INSPECT S50; FEI Company, Hillsboro, OR, USA, 200 nA current beam, −15 to 75° tilt, 360° rotation) under high vacuum conditions with magnification of 1000× and 5000× and aperture parameters from 4.5 to 5 at a working distance of 10 mm and electric power of 5 to 6 kilovolts [21,26].

### 2.6. Statistical Analysis

The descriptive analysis and the explanation of the database obtained were performed using a Microsoft Excel 2019 spreadsheet and the SPSS version 26 statistical program. Measures of central tendency and dispersion were calculated, including mean, standard deviation, standard error, median, interquartile range, and minimum and maximum values. For inferential analysis, the normality of the data was assessed using the Shapiro–Wilk test and the homogeneity of variances with Bartlett’s test. Student’s *t*-test (two-tailed) was applied to compare tooth enamel roughness and abrasion between the soft- and medium-filament brushing groups within each experimental treatment. Each control group was compared independently using the Student’s *t*-test, and the results are reported separately. A significance level of *p* < 0.05 was established. Statistical power was calculated using G*Power statistical software (version 3.1), considering a significance level of 0.05 (95% confidence).

## 3. Results

### 3.1. Abrasion and Roughness of Tooth Enamel

**Figure 3:**
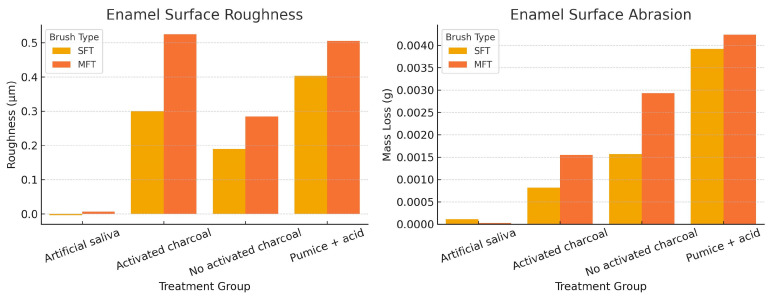
Comparison of enamel surface roughness (µm) and abrasion (g) after brushing cycles with soft (SFT) and medium (MFT) bristles, using toothpaste with and without activated charcoal. Greater roughness and abrasion are observed in the groups treated with activated charcoal toothpaste, especially with medium-bristle brushes.

Table 1 and Figure 3 shows the average changes in enamel roughness and abrasion in the groups treated with soft- and medium-bristle toothbrushes. Statistically significant differences were observed when using activated charcoal toothpaste, especially with medium bristles (*p* = 0.0016 for roughness and *p* = 0.0001 for abrasion). These findings indicate a possible increase in enamel wear under in vitro conditions. However, they should be interpreted within the experimental context and not directly extrapolated to the clinical setting.

### 3.2. Enamel Roughness According to Toothbrush and Toothpaste Type

Table 2 shows the mean, standard deviation, and median of the surface roughness values (μm). Under in vitro conditions, the highest mean roughness with soft-bristle brushes was observed in the group treated with pumice and acid (0.403 ± 0.222), followed by the group with activated charcoal toothpaste (0.300 ± 0.138). In the case of medium-bristle brushes, the highest mean and median values corresponded to the activated charcoal group (0.525 ± 0.134 and 0.502, respectively). These results indicate a possible trend toward greater enamel roughness with the use of more abrasive substances and stiffer bristles. However, their clinical implications should be considered with caution.

### 3.3. Enamel Abrasion According to Toothbrush and Toothpaste Type

Table 3 shows the mean, standard deviation, and median values corresponding to tooth enamel abrasion. Under in vitro conditions, the group treated with pumice and acid showed the highest abrasion values, both with soft filaments (−0.00392 ± 0.00144) and medium filaments (−0.00424 ± 0.00055). Among the experimental groups with toothpastes, the activated charcoal group showed higher abrasion values compared to the group without charcoal, especially with medium filaments (−0.00155 ± 0.00034 vs. −0.00293 ± 0.00275). Although the median values were similar between groups, there was a trend toward greater abrasion in the presence of more abrasive substances and stiffer filaments. However, as this was a controlled experimental setting, these findings should not be directly extrapolated to clinical practice.

### 3.4. Scanning Electron Microscope (SEM) Analysis of the Enamel Blocks

**Figure 4:**
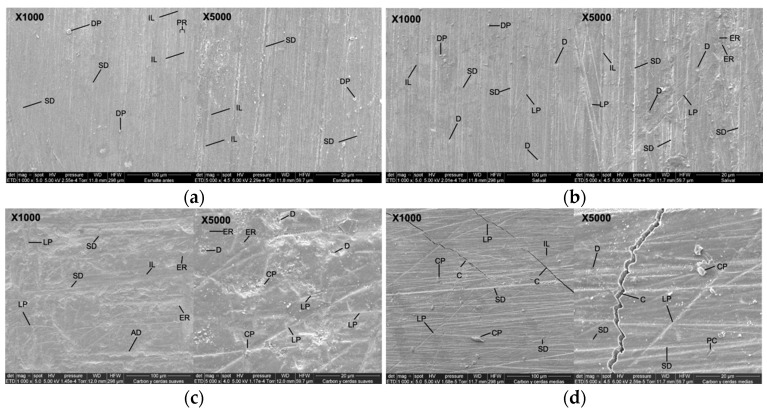

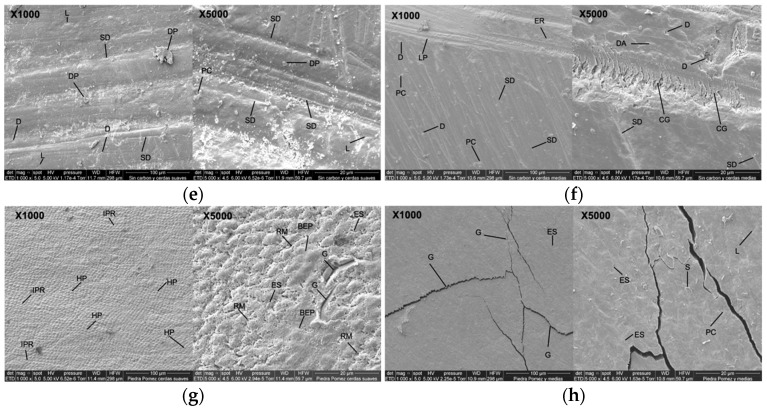
SEM micrographs of the surface of bovine enamel at 1000× and 5000× magnification. (**a**) Intact enamel with perichematics (PR), imbrication lines (IL), Retzius lines (SD), and superficial deposits (SD). (**b**) Artificial saliva: preserved surface with striae (SD), depressions (D), irregular lines (LP), enamel rod ends (ER), and DP. (**c**) Activated charcoal paste + brushing with soft bristles: enamel with ER, poorly defined IL, irregular SD, LP, D, and DA, and charcoal particles (CP). (**d**) Activated charcoal paste + brushing with medium bristles: deep SD and abundant CP, LP, PC, and C. (**e**) Paste without charcoal + brushing with soft bristles: unidirectional SD and D and regular L, DP, and PC. (**f**) Paste without charcoal + brushing with medium bristles: irregular ER, SD, LP, CG, DA, and PC. (**g**) Positive control (pumice stone + acid + brushing with soft bristles): severe abrasion, HP, IPR, RM, BEP, and G. (**h**) Positive control + brushing with medium bristles: poorly defined ES, S, PC, and extensive L and G. Abbreviations: PR: perichematics; IL: imbrication lines; SD: Retzius lines; DP: superficial deposits; ER: enamel rod ends; D: depressions; LP: irregular lines; CP: carbon particles; PC: craters; C: cracks; CG: crescent grooves; DA: depressogenic areas; HP: prism head; IPR: interprismatic region; RM: micro-retentions; BEP: prism body; ES: enamel sheath; G: grooves; S: fish scales; L: linear depressions.

SEM micrographs of the surface of bovine enamel at 1000× and 5000× magnification are presented in Figure 4. In Figure 4a (1000×), the uniform prismatic enamel is observed without any intervention, with imbrication lines (IL), perichematite ridges (PR), vertical striations of different depths (SD), and surface deposit particles (DP). At 5000×, the prismatic enamel showed remarkable variation, with imbrication lines (IL), striations of different depths (SD), and superficial deposits (DP).

Figure 4b (1000×) shows enamel that was immersed in artificial saliva with evidence of prismatic enamel and imbrication lines (IL), vertical striations of different depths (SD), lines that do not follow a pattern (LP), pitted depressions (D), and surface deposits (DP). At 5000×, enamel rod ends (ER), defined imbrication lines (IL), prominent vertical striated surfaces (SD), pit-like depressions (D), and lines that do not follow an increasing pattern (LP) are observed.

In Figure 4c (1000×), tooth enamel is observed after the brushing cycle with activated charcoal toothpaste using a soft-filament brush. Prismatic enamel is observed with poorly defined imbrication lines (IL), enamel rod ends (ER), striae of different depths (SD), lines that do not follow a pattern (LP), and depressed areas (DA). At 5000×, lines without any pattern (LP), pit-like depressions (D), charcoal particles (CP), and abundant enamel rod ends (ER) are observed.

Figure 4d (1000×) shows the dental enamel after the brushing cycle with toothpaste with activated charcoal and brushing using a medium-filament brush. The prismatic enamel is observed with striations of different depths (SD), scarce imbrication lines (IL), deposits and fragments of charcoal of greater size (CP), lines that do not follow a pattern (LP), and cracks (C). At 5000×, pronounced striations of different depths (SD), pits and craters of different sizes (PC), pit-like depressions (D), activated charcoal particles (CP), lines of irregular depth (LP), and cracks (C) are observed.

Figure 4e (1000×) shows the dental enamel after the brushing cycle with toothpaste without activated charcoal using a soft-filament brush. Scattered one-way striations with different depths (SD), pit-like depressions (D), defined and regular linear depressions (L), and superficial deposits (DP) are observed. At 5000×, there is widening of one-way and continuous striations (SD), pits and craters of different sizes (PC), defined linear depressions (L), and abundant superficial deposits (DP).

Figure 4f (1000×) shows the tooth enamel with a brushing cycle using toothpaste without activated charcoal and a medium-bristled brush; the ends of enamel rods (ER), irregular striations of different depths (SD), depressions that form pits (D), pits and craters (PC), and lines that do not follow the same pattern (LP) can be seen. At 5000×, slightly marked striations (SD), crescent-shaped perpendicular grooves (CG), pit-shaped depressions (D), and depressive areas (DA) can be seen.

In Figure 4g (1000×), the dental enamel is observed after the brushing cycle with pumice and etching acid using a soft-filament brush. The surface shows micro-retentions; the heads of the enamel prisms (HP) are evident and the interprismatic region (IPR) is defined. At 5000×, marked micro-retentions (RM), the affected enamel prism sheath (ES), the body of the enamel prism (BEP) where the prism head has been removed, and cracks and deep grooves (G) are evident.

In Figure 4h (1000×), the enamel is observed after the brushing cycle with pumice and etching acid using medium-filament brushes. The enamel prisms are almost completely removed; the prism sheath is poorly defined (ES) and shows some cracks and fractures (G). At 5000×, the prismatic surface of the enamel was observed with fish scale appearance (S), widening of the prism sheaths (ES), crater-like depressions (PC), and pits forming linear depressions (L).

## 4. Discussion

The characteristics of the tooth enamel surface play a fundamental role in its protective function, as they constitute the first line of defense against acids produced by bacteria, thus preserving the integrity of the dentin–pulp complex. It has been established that a surface roughness threshold of 0.2 μm favors the retention of microorganisms, which highlights the importance of maintaining a smooth surface to prevent bacterial adhesion and proliferation. Abrasion of the enamel can lead to a progressive and disproportionate loss of this tissue, which not only increases tooth sensitivity, but also increases the risk of caries and can compromise the structure of the pulp organ [11,12].

The results of the present study determined that there is a significant difference in enamel roughness (*p* = 0.0016) and abrasion (*p* = 0.0001) when using brushing cycles with activated charcoal toothpaste (Table 1). These findings are confirmed by the research conducted by Zamudio et al. and Maciel et al., who also indicated a significant difference in enamel abrasion when using toothpaste with activated charcoal (*p* < 0.05) [16,27]. The mechanical process caused by the constant friction of an object on the tooth surface is intensified with the use of larger particles, and this wear is further aggravated by vigorous brushing and the hardness of the brush filaments [28].

The roughness of the tooth enamel blocks that were brushed with medium filaments and toothpaste with activated charcoal presented a significantly higher mean value (0.525 ± 0.134). The variation of roughness in different groups also showed a significant difference (*p* = 0.0001); these results coincide with the studies of Forouzanfar et al. and Andrade et al., who determined that the difference in tooth enamel roughness was greater when using toothpaste with activated charcoal [22,29]. These results could be due to the fact that, in their methodology, a polishing process was used on the enamel blocks to obtain a smoother surface. The brushing cycles were performed with a cycling machine using linear and unidirectional movements.

It should be noted that activated charcoal has a rough and porous appearance, which, during brushing, could influence the superficial changes in the dental enamel due to the fact that these are larger insoluble abrasive particles, which act by mechanical and physical processes to remove extrinsic stains. In addition, the actual contact area is proportional to the load and pressure exerted, determined by roughness points, contributing to the increase in this roughness [3,18,30]. It should be noted that Lile I. et al. demonstrated that natural toothpastes, including those containing activated charcoal, can reduce plaque levels to clinically zero in a significant proportion of patients, provided that a controlled brushing technique is used. These findings reinforce the relevance of in vitro abrasion and roughness studies [31].

The present study evaluated dental abrasion through weight before and after the brushing cycles, demonstrating that the highest value of the mean weight difference was observed in the groups brushed with soft and medium filaments using toothpaste with activated charcoal. Similarly, a significant difference was observed when comparing brushing with medium filaments and toothpaste with activated charcoal (*p* = 0.01). Similar results were found by Greunling et al., who studied different types of toothpastes with activated charcoal and showed that one of these toothpastes presented a significantly higher difference in tooth enamel abrasion [6]; although the study performed the procedure using electric toothbrushes, these were connected to a cycling machine that simulated the brushing process with a frequency of 0.5 Hz. Similarly, Moreno et al. evaluated the abrasion of tooth enamel by the use of two toothpastes with activated charcoal and showed that both toothpastes caused weight loss with values of 4.11% and 2.68% [23]. These coincidences in the results could be attributed to the type of brush, with medium filaments. Viana I, et al. revealed that toothpastes with activated charcoal and the control group did not present a significant difference in surface loss and provided greater protection against abrasion [32]. In contrast to this research, in their study, they used optical profilometry and immersed the enamel blocks in citric acid solution for 5 min, followed by immersion in artificial saliva for one hour, repeating the process four times a day for 5 days. In the present study, a positive control group composed of an acid agent (etching acid) and an abrasive agent (pumice) was used, since both substances combined produce micro-abrasion. This is a technique used in the dental clinic to eliminate superficial alterations of the dental enamel, guaranteeing that there is a loss of up to 250 µm [23]. Although these results show statistically significant differences, it is essential to distinguish between statistical significance and clinical relevance. Given the in vitro nature of the study, the findings should not be directly extrapolated to clinical settings without caution.

Tooth enamel abrasion is significantly higher when brushing is performed with toothpaste containing activated charcoal, which adheres to extrinsic deposits such as pigmenting substances retained in the dental biofilm, through the pores of the charcoal, which are then eliminated by brushing, leaving the tooth surface free of these substances [3,17,31]. Although abrasion induced by activated charcoal toothpastes showed an increase, this does not necessarily imply a high clinical risk, since the behavior of enamel in the mouth responds to multiple biomechanical and biochemical variables.

The present investigation carried out the analysis of the samples through SEM; it should be noted that healthy dental enamel is formed by hydroxyapatite crystals and its structural unit is the enamel prism, and the external part of these prisms creates an interprismatic region [32]. Thus, the findings of the study (Figure 2) are in agreement with the results of Emídio et al., who show that, through the analysis of the micrographs, changes in the morphology of the dental enamel were observed in the groups treated with toothpaste with activated charcoal (Figure 2c,d) [14]. This is possibly due to the type of brush used and the brushing time, suggesting increased abrasion due to the stiffness of the brush filaments and the abrasive properties of the activated charcoal. These results differ from those observed in the study by Koc et al., who indicated that only a few scratches were observed on the enamel surface in all the groups studied [33]. This is possibly due to the fact that all groups in the study used toothpastes with activated charcoal and other abrasive particles.

The morphology of the enamel in the microphotographs obtained in this study was observed with greater alteration when the brushing process was performed with medium filaments and paste with activated charcoal (Figure 2d), with disorganized striations being visible, indicating increased surface roughness.

Several studies support the use of bovine teeth as viable substitutes for human teeth in in vitro research due to their structural and physicochemical similarities. They have been shown to have comparable values for radiodensity, enamel thickness, and microhardness, as well as a similar histological organization with similar dentin characteristics. These conditions make bovine enamel an accepted model for studies of wear, abrasion, and adhesion, given the minimal microscopic and macroscopic differences from human tissue [34,35].

Although this in vitro study provides relevant findings on the abrasive effects of activated charcoal toothpaste, certain methodological limitations should be considered when interpreting the results. No corrections were made for multiple comparisons due to the exploratory nature and small sample size, which could increase the risk of type I error. The brushing simulator used a unidirectional movement, which does not fully reflect the kinematic complexity of human clinical brushing. Furthermore, only two toothpastes were evaluated, without including a more diverse range of commercial products. Finally, the simulated brushing protocol represented short-term exposure (approximately 20 days), which limits the extrapolation to long-term cumulative effects. These considerations do not invalidate the findings obtained, but they highlight the need for future studies with greater external validity and simulations that are closer to the clinical environment.

## 5. Conclusions

The findings obtained in this study allowed us to reject the null hypothesis, as statistically significant differences were found in the levels of abrasion and roughness of tooth enamel depending on the type of toothpaste (with or without activated carbon) and the type of toothbrush (soft and medium filaments).

This in vitro study showed that the use of activated charcoal toothpaste increases the abrasion and roughness of tooth enamel, particularly when medium-filament toothbrushes are used. These findings were reflected in the morphological alterations observed by scanning electron microscopy. Although statistically significant differences were identified in some groups, the results should be interpreted with caution due to the limitations of the experimental design, such as the absence of real clinical conditions and the small sample size. Based on the results obtained, it is suggested that frequent use of activated charcoal toothpastes in combination with medium-filament toothbrushes be limited until more extensive and representative clinical studies confirm their safety. It is also recommended that these findings be validated through future research that integrates clinical simulations and greater standardization of variables.

## Figures and Tables

**Table 1:** Abrasion and roughness of tooth enamel on brushing using toothpaste with and without activated charcoal.

		Roughness			Abrasion		
Variable	n	SFT	MFT	*p*-Value	SFT	MFT	*p*-Value
Artificial saliva	10	−0.0037	0.0067	0.1292	−0.00011	−0.00003	0.2400
Activated charcoal	10	0.29963	0.5251	0.0016 *	−0.00082	−0.00155	0.0001 *
No activated charcoal	10	0.1895	0.2847	0.0971	−0.00157	−0.00293	0.5188
Pumice stone and acid	10	0.4034	0.5052	0.2522	−0.00392	−0.00424	0.5186

Student’s *t*-test. * *p* < 0.05 significant. SFT: soft-filament toothbrush; MFT: medium-filament toothbrush.

**Table 2:** Difference in tooth enamel roughness (μm) before and after using a toothbrush with soft and medium filaments using toothpaste with and without activated charcoal.

		**Roughness (μm) with Soft-Filament Toothbrush**						
**Groups**	**n**	**(** $\bar{x_{f}}$ ** − ** $\bar{x_{i}}$ **)**	**S.D.**	**SE**	**Median**	**IQR**	**Min.**	**Max.**
Artificial saliva	10	−0.004	0.018	0.006	−0.001	0.027	−0.038	0.014
Activated charcoal	10	0.300	0.138	0.044	0.282	0.194	0.131	0.596
No activated charcoal	10	0.189	0.085	0.027	0.208	0.158	0.071	0.330
Pumice stone and acid	10	0.403	0.222	0.070	0.387	0.366	0.123	0.802
		**Roughness (μm) with Medium-Filament Toothbrush**						
**Groups**	**n**	**(** $\bar{x_{f}}$ ** − ** $\bar{x_{i}}$ **)**	**D.E**	**EE**	**Median**	**IQR**	**Min.**	**Max.**
Artificial saliva	10	0.007	0.010	0.003	0.008	0.011	−0.013	0.025
Activated charcoal	10	0.525	0.134	0.042	0.502	0.182	0.376	0.841
No activated charcoal	10	0.285	0.150	0.047	0.272	0.191	0.110	0.619
Pumice stone and acid	10	0.505	0.157	0.050	0.468	0.259	0.321	0.764

n: sample size; ($\bar{x_{f}}$ − $\bar{x_{i}}$): mean surface roughness; $\bar{x_{f}}$: after; $\bar{x_{i}}$: before; S.D.: standard deviation; SE: statistical error; IQR: interquartile range.

**Table 3:** Difference in tooth enamel abrasion before and after using a toothbrush with soft and medium filaments using toothpaste with and without activated charcoal.

		**Abrasion with Soft-Filament Toothbrush**						
**Groups**	**n**	**(** $\bar{x_{f}}$ ** − ** $\bar{x_{i}}$ **)**	**S.D.**	**SE**	**Median**	**IQR**	**Min.**	**Max.**
Artificial saliva	10	−0.00011	0.00013	0.00004	−0.00010	0.00020	−0.0004	0
Activated charcoal	10	−0.00082	0.00025	0.00008	−0.00090	0.00038	−0.0011	−0.0004
No activated charcoal	10	−0.00157	0.00065	0.00021	−0.00130	0.00062	−0.0032	−0.0010
Pumice stone	10	−0.00392	0.00144	0.00045	−0.00380	0.00180	−0.0068	−0.0021
		**Abrasion with Medium-Filament Toothbrush**						
**Groups**	**n**	**(** $\bar{x_{f}}$ ** − ** $\bar{x_{i}}$ **)**	**D.E**	**EE**	**Mediana**	**IQR**	**Min.**	**Max.**
Artificial saliva	10	−3 × 10^−5^	0.00016	0.00005	−0.00010	0.00020	−0.0004	0.00003
Activated charcoal	10	−0.00155	0.00034	0.00011	−0.00090	0.00038	−0.0021	−0.0011
No activated charcoal	10	−0.00293	0.00275	0.00087	−0.00130	0.00062	−0.0094	−0.001
Pumice stone	10	−0.00424	0.00055	0.00017	−0.00380	0.00180	−0.0052	−0.0035

n: sample size; ($\bar{x_{f}}$ − $\bar{x_{i}}$): abrasion mean; $\bar{x_{f}}$: after; $\bar{x_{i}}$: before; S.D.: standard deviation; SE: statistical error; IQR: interquartile range.

## Data Availability

The data supporting this study’s findings are available from the corresponding author upon reasonable request.
